# Potential categories of employment stress among rural college students and their relationship to employment psychology

**DOI:** 10.3389/fpsyg.2024.1363065

**Published:** 2024-03-28

**Authors:** Xinyue Wu, Kyung Yee Kim, Ziting Jian

**Affiliations:** ^1^Student Affairs Department, Qingdao University of Technology, Qingdao, China; ^2^Department of Education, General Graduate School, The Catholic University of Korea, Bucheon-si, Republic of Korea

**Keywords:** employment psychology, rural students, job-seeking stress, intervention programs, potential categories

## Abstract

**Background:**

Psychological problems related to employment are among the most common psychological problems faced by rural college students. Employment stress is an important factor affecting the development of psychological health in employees; thus, reducing employment stress can improve the psychological state of employment.

**Objective:**

This study aimed to understand the potential profiles of employment stress among rural college students to determine the relationship between different profiles and employment psychology.

**Methods:**

This study was conducted in a higher education institution in Qingdao, Shandong Province, China between June and December 2023, and 249 rural college students participated. The Employment Stress Scale and Employment Psychology Scale were used to collect the data. Data were analyzed using latent profile analysis, independent sample *t*-tests, and binary logistic regression analysis.

**Results:**

The results showed that rural university students were categorized into low-level (49.80%) and high-level (50.21%) employment stress groups. There was a statistically significant difference between the employment psychology of rural college students in the low- and high-level groups (*p* < 0.001). Juniors/seniors were more likely to be classified in the high-level group (OR = 0.477, *p* = 0.011).

**Conclusion:**

Intervention programs should be developed and implemented to address the characteristics of employment stress among rural college students with different profiles to promote the healthy development of their attitudes toward employment.

## 1 Introduction

Statistics from China’s Ministry of Education of the People’s Republic of China (n.d.) showed that the number of college students in China was 30.32 million in 2019, and as of 2022 had risen as high as 36.56 million, with rural college students accounting for about 47% of the total. The above data is evidence of the high growth rate in the number of college students in China in recent years, and the number of employed people is also increasing (Senekal and Smith, 2022; Yin, 2022). However, there is no large-scale postgraduate expansion and recruitment program in China, which leads to a grim employment situation for college students (Pei, 2021; Li, 2022; Li et al., 2022; Xiong et al., 2022; Zhi, 2023). This phenomenon causes college students to adapt to their current form of employment; however, the process of adaptation produces complex and fluctuating psychological changes related to employment. All of these factors are usually referred to as “employment psychology” (Achdut and Refaeli, 2020; Wei, 2023).

Employment psychology plays an important role in the lives, studies, employment, and career choices of college students (Hernández-Sánchez et al., 2020). Previous studies have shown that good employment psychology can help mobilize college students’ strong learning motivation and improve their comprehensive ability to adapt to the employment environment in China (Luciano et al., 2021; Senekal and Smith, 2022). In contrast, poor employment psychology affects college students’ thinking patterns and memory and reduces their employment competitiveness (Jackson and Tomlinson, 2020; Wan Mohd Yunus et al., 2021; Peng et al., 2023). In summary, good employment psychology is of great significance in promoting the successful employment of rural college students.

However, the results of the current study show that the employment psychological level of college students is generally not high (Liu et al., 2019; Saddik et al., 2020; Song et al., 2021), and rural college students are more prone to negative psychological factors, such as low self-esteem and anxiety due to the heavy economic burden carried by their families, leading to low self-evaluation and a narrowing of the scope of employment (Chen et al., 2020; Pillay et al., 2020). In addition, most parents of rural college students have a low level of education, lack appropriate parenting styles, and provide insufficient emotional support, which leads to difficulties in regulating the emotions of rural college students and a poor employment mentality, diminishing the effects of employment. Therefore, it is important to explore the potential factors influencing rural college students’ employment psychology to enhance their employability and help them succeed.

According to social resources theory, scarcity of social resources leads to great stress and affects the mental health of individuals (Brunner et al., 2019; Goldmann, 2019). Employment stress refers to a series of physiological, psychological, and behavioral response processes that arise from internal and external stressors during an individual’s job search and selection (Ganster and Rosen, 2013; Wong et al., 2021). For rural college students in the process of seeking employment, there are problems such as the poor job market, poor employability, and insufficient social support (Mishra, 2020). These many sources of employment-related pressure result in enormous pressure, representing a major component in their employment psychology makeup (Arias-de la Torre et al., 2019).

Lee (2020) research showed a negative correlation between employment stress and employment psychology in a group of rural college students. Compared with rural college students with a high level of employment stress, rural college students with a low level of employment stress have a more objective personal evaluation, a better outlook on employment, and a healthier employment psychology. These results suggest that alleviating employment pressure on rural college students may be an important focus for improving their employment psychology. However, most studies have assessed the employment stress levels of rural college students only by evaluating their total employment stress scores, ignoring the group heterogeneity of rural college students’ employment stress, and therefore these results cannot provide precise suggestions for the development and implementation of relevant interventions.

Previous studies have shown that employment stress includes many aspects such as personal factors, social environment, family and friends, school, and professional issues, and that rural college students have different characteristics of employment stress when they face different employment dilemmas (Szromek and Wolniak, 2020; Kossek et al., 2021; Ojo et al., 2021; Rawal, 2023). According to the characteristics of the employment pressure on rural college students, formulating individualized employment guidance and support will help adjust their employment psychology and employment outlook, and effectively help rural college students achieve successful employment (Li et al., 2022). Previous studies showed that rural college students with the same employment psychology presented different employment stress characteristics (Belle et al., 2022; von Keyserlingk et al., 2022). Therefore, correctly assessing the employment stress characteristics of rural college students can guide researchers in developing individualized intervention strategies to promote their employment-related mental health.

Potential profile analysis classifies individuals into potential profiles based on the intersection of multiple features of the observed variables (Marsh et al., 2009; Lanza and Rhoades, 2013; Yang Q. et al., 2022). Thus, latent profile analysis can categorize rural college students with the same employment stress traits and characteristics into similar categories, ensuring intergroup heterogeneity among rural college students. Previous studies have analyzed stress, positive thoughts, anxiety, and needs using latent profile analysis with college students as research subjects. However, potential profiling of categories of employment stress among rural college students has not yet been conducted. Therefore, this study aims to identify the potential profiles of employment stress among rural college students, explore the factors affecting the classification of employment stress subgroups in terms of demographic characteristics, compare the employment psychology of rural college students in different employment stress subgroups, and provide targeted suggestions to induce an appropriate employment psychology.

## 2 Methods

### 2.1 Participants

A total of 254 rural college students enrolled in a higher education institution in Qingdao, Shandong Province, China, ranging from freshmen to seniors, were selected from August to November 2023 using convenience sampling. The inclusion criteria were: no mental disorders, informed consent, voluntary participation, and good physical health. The exclusion criteria were being under 18 years of age and not likely to seek employment. Of the 254 rural college students initially identified for research, 5 were excluded due to 20% or more of the questionnaire remaining unanswered and obvious patterns, giving a final total of 249 research subjects (67.9% female), an effective questionnaire recovery rate of 0.98.

### 2.2 Research process

The process was approved by the Bioethics Review Committee of Sacred Heart Campus of The Catholic University of Korea (CUK-IRB) (Institutional Review Board) (Number: 1040395–202,312-031). By reading the records of one-on-one heart-to-heart conversations among college students and checking the information in the students’ files, the research subjects eligible for this study were included. The help of the school leaders was sought to explain the purpose and significance of this study, to inform the students of the voluntary nature of the survey, and assure them the relevant information would be kept strictly confidential. Verbal consent was then received from the student participants. Relevant questionnaires were then collected by counselors and classroom teachers, and each questionnaire took approximately 25–30 min to complete.

## 3 Instruments

### 3.1 The demographic questionnaire

The General Demographic Information form was used to collect demographic characteristics of rural college students, and gathered basic information such as gender, age, grade level, and whether or not they were student leaders.

### 3.2 Employment stressors questionnaire

The Employment Stressor Scale utilizes the Employment Stressor Moderation Questionnaire developed by Dai (2009). This employment stressor scale has been widely used in China and has been shown to have good reliability. The Employment Stressors Scale consists of five parts: personal factors (five items), social environment factors (four), family and friends factors (four), school factors (three), and professional factors (five), for a total of 21 items. Each item was rated on a 5-point Likert scale ranging from “1 (not at all compliant)” to “5 (fully compliant).” The total score ranged from 21 to 105, with higher scores indicating higher levels of employment stress. The Cronbach’s alpha coefficient for this scale in this study was 0.812.

### 3.3 Employment psychology questionnaire

The Employment Psychology Scale was adopted from Liu (2010) self-edited Employment Psychology Questionnaire for College Students. The Employment Psychology Scale has been validated for research use with Chinese groups and consists of four parts: Preparation for Employment (12 items), Employment Perceptions (10), Employability (8), and Employment Perceptions (7), giving 37 items in total. Each item was rated on a 5-point Likert scale ranging from “1 (not at all compliant)” to “5 (fully compliant).” The total score ranged from 37 to 185, with higher scores representing a greater psychological likelihood of employment. Cronbach’s coefficient for this scale in this study was 0.763.

### 3.4 Data analyses

Potential profiles were analyzed using Mplus 7.0. From the initial one-profile model to the final four-profile model, all the parameters were estimated by gradually increasing the number of profiles until the best-fit index emerged. The Akaike Information Criterion (AIC) and Bayesian Information Criterion (BIC) were used to identify the optimal model, followed by Lo–Mendell–Rubin (LMR) *p*-values and Bootstrap Likelihood Ratio Test (BLRT) *p*-values to determine whether the former profile was the best-fitting model. The specific criteria were as follows: smaller AIC and BIC values implied a better model fit. The values of LMRT (p) and BLRT (p) were less than 0.05, implying that the previous profile was the best-fitting model. In addition, entropy values greater than 0.8 meant that the potential profiles were well separated.

Other data analyses were performed using the IBM SPSS software (version 25.0). The results of the histogram and Q-Q plot analyses showed that the data in this study conformed to an approximately normal distribution. After determining the optimal model, mean ± standard deviation was used to describe the employment stress scores. Independent sample *t*-tests were conducted to compare the psychological differences in employment experienced by participants across potential profiles. The relationship between the factors affecting the potential employment pressure profile and demographic characteristics was explored using chi-square tests and a multiple regression analysis.

## 4 Results

### 4.1 Latent profiles of employment stress

This study analyzed models with different numbers of profiles and compared four models. The AIC and BIC of the two-profile model are significant as well as entropy values greater than 0.8 for the two-profile model, although they are not minimized, for the LMRT(p) and BLRT(p) values. The LMRT(p) values were not significant when comparing the two- and three-profile models, indicating that the three-profile model was not superior to the two-profile model. Table 1 presents the model fit statistics for the potential profile estimates.

**Table 1:** Results of applying the potential profile model of employment pressure on rural college students (*N* = 249).

Latent Profile	AIC	BIC	SSA-BIC	Entropy	LMRT(p)	BLRT(p)	Smallest class (%)
One-class	6603.309	6638.484	6606.783	–	–	–	–
Two-class	6179.316	6235.595	6184.874	0.845	**<0.001**	<**0.001**	49.40
Three-class	6053.951	6131.335	6061.594	0.809	0.441	<0.001	20.88
Four-class	5994.209	6092.697	6092.697	0.825	0.438	<0.001	8.84

-, not applicable. Akaike Information Criterion (AIC), Bayesian Information Criterion (BIC), Sample Size-Adjusted BIC (aBIC), Lo–Mendell–Rubin (LMR), and Bootstrap Likelihood Ratio Test (BLRT). Text in bold indicates the lowest BIC, AIC, and aBIC values. *P-values for LMRT and BLRT were <0.05.

**Figure 1:**
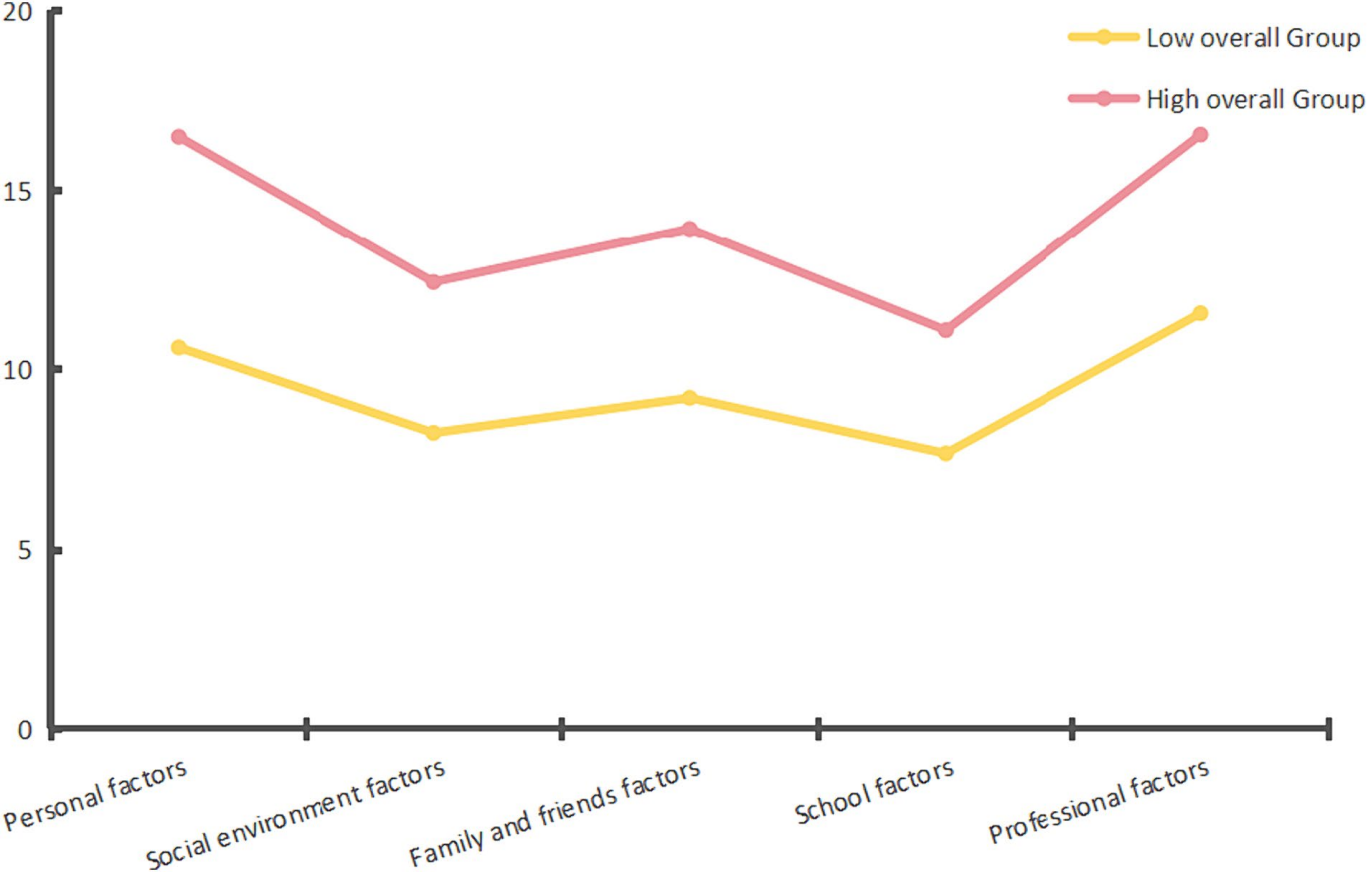
Line graph of mean scores for each dimension of the two potential profiles of employment stress among rural college students.

Each profile was named based on the score for each dimension of employment stress. The results of this study show a significant difference in employment stress dimension scores between the two subgroups of rural college students. Consequently, the two employment stress profile model categories were designated as high- and low-level groups. This is shown in Table 2 and Figure 1.

**Table 2:** Difference-in-differences analysis of scores on dimensions of two potential profiles of employment stress among rural college students (*N* = 249).

Study variable	Low overall group (*N* = 123)		High overall group (*N* = 126)		*t*	*P*
	*M*	*SD*	*M*	*SD*		
Personal factors	10.62	2.74	16.5	3.01	−16.114	<0.001
Social environment factors	8.25	1.87	12.44	2.2	−16.163	<0.001
Family and friends factors	9.22	2.57	13.94	2.8	−13.887	<0.001
School factors	7.68	2.19	11.1	1.85	−13.301	<0.001
Professional factors	11.57	3.2	16.56	3.17	−12.382	<0.001

Profile 1 (high overall group, 49.4%) had a high probability of occurrence for almost all the items. Compared to Profile 2, Profile 1 scored higher on personal, social environment, family and friends, school, and professional issues. This indicated that this group of rural college students experienced high levels of employment stress. Profile 2 (low overall group: 50.6%) had a low probability of appearing at a low level for almost all items. This indicates that this group of rural college students experienced a low level of employment pressure.

### 4.2 Differences in employment psychology

As shown in Table 3, there were statistically significant differences between the two groups in terms of employability (*t* = 3.969, *p* < 0.001), employment cognition (*t* = 2.272, *p* = 0.024), and employment psychology (*t* = 2.101, *p* = 0.037), and the differences were statistically significant.

**Table 3:** Differences in the level of employment psychology among rural college students in different employment stress groups (*N* = 249).

Study variable	Low overall group		High overall group		*t*	*P*
	(*N* = 123)		(*N* = 126)			
	*M*	*SD*	*M*	*SD*		
Employment preparation	45.47	4.94	44.81	4.58	1.098	0.273
Employment concepts	39.18	4.69	39.79	5.52	−0.934	0.351
Employability	31.13	4.31	28.92	4.47	3.969	<0.001
Employment cognition	28.58	2.88	27.7	3.21	2.272	0.024
Employment psychology	144.36	11.41	141.21	12.18	2.101	0.037

### 4.3 Differences in characteristics

As shown in Tables 4, 5, there was a significant difference between the two groups of rural college students at the grade level (χ2 = 4.997, *p* = 0.025), with juniors and seniors more likely to be in the high-level group (OR = 2.134, CI = 1.157–3.93, *p* = 0.015).

**Table 4:** Differences in demographic distribution of employment stress types among rural college students in different employment stress groups (*N* = 249).

Characteristics		Low overall group (*n* = 123)	High overall group (*n* = 126)	χ^2^	*P*
Type of discipline	Arts	55 (44.7%)	52 (413%)	0.302	0.583
	Science	68 (55.3%)	74 (58.7%)		
Gender	Male	40 (32.5%)	40 (31.7%)	0.017	0.896
	Female	83 (67.5%)	86 (68.3%)		
Grade	Freshman/Sophomore	23 (18.7%)	39 (31.0%)	4.997	0.025
	Junior/Senior	100 (81.3%)	87 (69.0%)		
Whether an only child	Yes	19 (15.4%)	24 (19.0%)	0.565	0.452
	No	104 (84.6%)	102 (81.0%)		
Whether student leader	No	17 (13.8%)	21 (16.7%)	0.390	0.532
	No	106 (86.2%)	105 (83.3%)		
Choice after graduation	Employment	61 (49.6%)	57 (45.2%)	0.474	0.491
	Continuing education	62 (50.4%)	69 (54.8%)		

**Table 5:** Regression analysis of employment stress characteristics of rural college students across potential profiles (*N* = 249).

Characteristics	*B*	Standard error	Significance	Exp(B)	EXP(B) 95% confidence interval	
					Upper	Lower limits
Constant	−0.077	0.248	0.754	0.925		
Subject type (science)	−0.208	0.271	0.443	0.812	0.478	1.381
Sex (female)	−0.078	0.307	0.798	0.925	0.507	1.687
Academic background (junior/senior)	0.758	0.312	0.015	2.134	1.157	3.935
Only child (no)	0.304	0.367	0.407	1.356	0.66	2.784
Whether student leader (no)	0.348	0.372	0.349	1.417	0.684	2.936
Choice after graduation (continuing education)	−0.158	0.265	0.551	0.854	0.508	1.436

## 5 Discussion

This study not only explored the employment stress characteristics of rural college students but also investigated potential employment stress categories and the relationship between those stress categories and employment psychology. Ultimately, the results of this study indicate that rural college students can be categorized into two employment stress subgroups and that these subgroups can provide directions for intervention and points of focus for future research.

The results of the potential profile analysis highlight significant individual differences in the employment pressure of rural college students in this study, which can be divided into a low-level group (Category 1) and a high-level group (Category 2), accounting for 49.40 and 50.60% of the total number, respectively, indicating serious polarization in the employment pressure of rural college students.

The reasons for this may be related to different individual perceptions of employment opportunities. For example, Yang S. et al. (2022) showed that the more sensitive the perception of employment opportunities, the greater the individual’s employment stress. Therefore, colleges, universities, and recruiters should communicate and exchange more information to convey the real employment situation to rural college students and help them establish correct perceptions of employment opportunities.

The results showed that those in the high level group had higher scores for personal factors, social environment, family and friends, school, and professional issues, suggesting that employment stress among rural college students is the result of a combination of factors. This result is consistent with the findings of Li et al. (2022), whose study showed that personal and professional factors are related to students’ personal evaluations and employment expectations, and that low personal evaluations and high employment expectations often lead to a loss of individual self-confidence in employment and a sudden increase in employment pressure. In addition, according to the stimulus-physiological/psychological-response (SOR) theoretical model, external factors can produce a direct psychological response in the body: when the employment social environment the is highly competitive, there are too few jobs, and the school’s previous students have poor employment records, it will increase the individual’s employment panic (Ardern et al., 2012; Anselme and Güntürkün, 2019; Mladenović et al., 2023).

Salami et al. (2021) showed that family support was strongly related to individuals’ employment stress. This study suggested that social support from family and friends increases college students’ sense of security and reduces negative emotions. In addition, the contrast between the high expectations of the parents of rural college students and the currently highly competitive employment environment may also increase employment pressure on these students.

Another factor is that the economic conditions in which the families of rural college students exist tend to be, at best, average, and raising a college student exhausts most of the family’s resources; parents therefore expect their children to find a good job and help their families, which leads to great pressure on rural college students to find employment.

Based on these factors, schools should encourage students to establish a correct view of career choices, change the traditional concept of employment, give full play to their own strengths, and choose suitable employment positions. Teachers should organize class meetings with outstanding graduates to enhance rural college students’ confidence in employment. Simultaneously, an integrated school-family training model should be established to mobilize the emotional support of family and friends for these students.

The results also show that gender and being a student leader have no effect on employment pressure, mainly because the trend of rural college students choosing to go to higher education has gradually become more prominent, and the number of students going to graduate school has been increasing annually, while the number of urban students has been decreasing. This phenomenon shows that, although an urban–rural gap still exists, the differences in job characteristics in the primary and secondary labor markets are widening, and rural students dominate the examination group. Therefore, the influence of student cadres and gender factors on rural university students’ employment pressure is relatively small, and the main factors still lie in the rural economy and the structural problems of the labor market.

The results also highlighted the difference between the low- and high-level groups on the total scores of Employability, Employment Perceptions, and Employment Psychology (Lee, 2020; Yang S. et al., 2022), which is consistent with the studies of Lee. On the one hand, this may be due to the fact that rural college students under significant pressure to find employment will be immersed in negative emotions and adopt negative coping methods, such as implicit learning and avoidance, to deal with this pressure. This ultimately leads to reduced learning input behavior, decreased employability, and increased negative emotions.

The fact that most rural college students are first-generation college students in their families, with relatively fewer social resources available and less employment information and opportunities can cause them to be picky about employment positions and blindly follow current trends and exhibit other behaviors during the employment process, which affects employment cognition and leads to poor employment psychology. In summary, schools should pay attention to providing psychological guidance for rural college students, open public psychological counseling rooms, and effectively monitor the psychological condition of rural college students. They should establish an effective employment training system, carry out timely employment guidance, help rural college students set up correct employment goals, and provide effective employment assistance.

A key aspect of the results was the identification of grade level as an important indicator for identifying high employment stress among college students. This study found that rural junior/senior college students were more likely to fall into the high-level group, which is consistent with the findings of previous research. This may be due to the fact that juniors/seniors are at the fork of employment and further education and are directly faced with the problem of solving the employment problem, making employment pressures relatively greater. There is also the fact that after apprenticeships and internships, junior and senior students have a clearer understanding of the vocational skills required for employment positions, while their own professional knowledge is not yet solidly grasped, leading fear of being rejected for employment by employers.

Previous studies have found gender differences in employment stress and indicated that women experience higher levels of employment stress than men because they are more sensitive and perceive stress more easily, while men are more resilient to setbacks and have greater mental toughness in withstanding the psychological stress caused by factors related to employment (Juster et al., 2019; Christiansen and Berke, 2020; Debowska et al., 2022). In addition, Wang’s research showed that the employment pressure on college students who are only children is often lower than that on students from larger families, due to the fact that one-child families can utilize all the family resources of the family and devote more parental attention and care to the child. This gives such children more sources of social support and helps to alleviate the employment pressure (Chang et al., 2020; Islam et al., 2020; Owens et al., 2020). On the other hand, children with siblings who have to solve employment problems on their own and often bear the responsibility of taking care of those siblings are more eager and under pressure to obtain employment, which increases their stress. However, while data was gathered on gender and one-child v. multi-child families, neither issue was included in the regression equation model in this study. In relation to gender. For the issue of whether participants were from one-child or multi-child families, the issue was omitted because of the relatively proportion number of women, who almost always have siblings, and the relatively small sample size of men, who form the majority of only children (Chow et al., 2019; Goodman, 2020).

This study considered the heterogeneity of rural college students; the potential categories of employment stress among rural college students were explored using potential profile analysis, and their relationships with psychological and sociodemographic employment profiles were analyzed, which provided a theoretical basis for the development of targeted psychological interventions. However, this study has several limitations. First, it examined only rural college students at a single higher education institution in Shandong Province. This implies a possible selection bias because rural development is uneven across regions in China; employment pressure may therefore vary. Future research should investigate the current status of employment stress among rural college students in multiple regions and colleges to increase sample diversity. Second, this study did not fully consider the effect of covariates when conducting LPA; therefore, subsequent studies should incorporate additional covariates based on relevant theories to ensure grouping accuracy.

## 6 Conclusion

This study analyzed the characteristics of employment stress among rural college students through potential profile analysis and identified a low-level group (Category 1) and a high-level group (Category 2). This study found differences in employment psychology between the two groups. Grade level as a risk factor for employment stress among rural college students. In conclusion, the different categorization characteristics of rural college students’ employment stress can help schools identify potential high-risk groups at an early stage and provide timely and targeted interventions. Such interventions can have beneficial effects and effectively alleviate the employment stress of rural college students.

## Data availability statement

The raw data supporting the conclusions of this article will be made available by the authors, without undue reservation.

## Ethics statement

The studies involving humans were approved by Bioethics Review Committee of Sacred Heart Campus of The Catholic University of Korea (CUK-IRB) (Institutional Review Board) (Number: 1040395-202312-031). The studies were conducted in accordance with the local legislation and institutional requirements. The participants provided their written informed consent to participate in this study.

## Author contributions

XW: Conceptualization, Data curation, Formal analysis, Investigation, Methodology, Software, Validation, Writing – original draft, Writing – review & editing. KK: Formal analysis, Project administration, Supervision, Writing – review & editing. ZJ: Data curation, Software, Writing – review & editing.
